# Brain structure abnormalities in young women who presented conduct disorder in childhood/adolescence

**DOI:** 10.3758/s13415-017-0519-7

**Published:** 2017-07-10

**Authors:** Meenal Budhiraja, Ivanka Savic, Philip Lindner, Jussi Jokinen, Jari Tiihonen, Sheilagh Hodgins

**Affiliations:** Department of Clinical Neuroscience, Karolinska University Hospital, Psychiatry Building R5:00, 171 76 Stockholm, Sweden

**Keywords:** Females, Conduct disorder, Brain, Magnetic resonance imaging, Gray matter volume

## Abstract

The phenotype and genotype of antisocial behavior among females are different from those among males. Previous studies have documented structural brain alterations in males with antisocial behavior, yet little is known about the neural correlates of female antisocial behavior. The present study examined young women who had presented conduct disorder (CDW) prior to age 15 to determine whether brain abnormalities are present in adulthood and whether the observed abnormalities are associated with comorbid disorders or maltreatment that typically characterize this population. Using magnetic resonance imaging and voxel-based morphometry, we compared gray matter volumes (GMV) of 31 women who presented CD by midadolescence and 25 healthy women (HW), age, on average, 23 years. Participants completed structured, validated interviews to diagnose mental disorders, and validated questionnaires to document physical and sexual abuse. Relative to HW, CDW presented increased GMV in the left superior temporal gyrus that was associated with past alcohol and drug dependence, current use of alcohol and drugs, and current anxiety and depression symptoms and maltreatment. Additionally, CDW displayed reduced GMV in lingual gyrus, hippocampus, and anterior cingulate cortex that was associated with past comorbid disorders, current alcohol and drugs use, current anxiety and depression symptoms, and maltreatment. The CDW also presented reduced total GMV that was associated with past comorbid disorders and current anxiety/depression symptoms. Alterations of brain structure were observed among young adult females with prior CD, relative to HW, all of which were associated with internalizing and externalizing disorders and maltreatment that typically accompany CD.

Conduct disorder (CD) affects between 0.8% and 9.2% of girls prior to age 15 (Loeber, Burke, Lahey, Winters, & Zera, 2000). These girls are at increased risk for mental disorders, criminality, dysfunctional and often violent relationships, unemployment, and premature mortality (Odgers et al., 2008). Further, they tend to have babies at a young age (Jaffee, 2002) who are at high risk for antisocial behavior themselves (D’Onofrio et al., 2007; Jaffee, Belsky, Harrington, Caspi, & Moffitt, 2006). Just as clinical services are confronted by teenage girls engaging in antisocial and aggressive behavior, who typically also present anxiety and depression disorders and a history of maltreatment, neuroscience is challenged to unravel the neural mechanisms associated with these multiple disorders and traumas that have the potential to derail healthy brain development.

The neuroanatomical correlates of CD and its adult sequelae, antisocial personality disorder (ASPD), have been investigated using magnetic resonance imaging (MRI), primarily in males. Findings converge in identifying abnormalities of gray matter volume (GMV) in the frontal and temporo-limbic structures among men with ASPD (Dolan, Deakin, Roberts, & Anderson, 2002; Glenn, Raine, Yaralian, & Yang, 2010; Gregory et al., 2012; Müller et al., 2008; Raine, Lencz, Bihrle, LaCasse, & Colletti, 2000; Tiihonen et al., 2008) and adolescent boys with CD (Dalwani et al., 2011; De Brito, Mechelli, Wilke, et al., 2009; Fairchild et al., 2011; Huebner et al., 2008; Kruesi, Casanova, Mannheim, et al., 2004; Sterzer, Stadler, Poustka, et al., 2007). The samples included in these studies varied considerably as to aggressive behavior, criminality, psychopathic traits, whether or how substance misuse was measured and controlled in statistical analyses, and most did not assess comorbid mental disorders or maltreatment.

The phenotype and genotype of CD among females differ from those among males. CD is less prevalent among girls than boys. In childhood, four times more boys than girls present CD (Maughan, Rowe, Messer, Goodman, & Meltzer, 2004), and in adolescence, twice as many boys as girls (Loeber et al., 2000). The presentation of CD also differs among girls and boys (Lahey et al., 2006). CD in females is characterized by a later age of onset, a distinct developmental course, and less aggressive behaviour (Brennan & Shaw, 2013; Gelhorn et al., 2009). Further, girls tend to engage in relational aggression while boys are more likely to show physical aggression (Kroneman, Loeber, Hipwell, & Koot, 2009). Females with CD are also more likely than males with CD to exhibit comorbid mental disorders (Costello, Foley, & Angold, 2006). Additionally, females with CD are exposed earlier than their peers to alcohol and drugs and most go on to misuse these substances (Odgers et al., 2008). Evidence is accumulating to show that not only the CD phenotype but the genotype may also vary by sex. Genetic factors have been shown to explain more variance of CD in females than in males (D’Onofrio et al., 2007). Further, among females it is the interaction of the high acting variant of Monoamine-oxidase A gene, not the low acting variant as in males (Byrd & Manuck, 2014; Sjöberg et al., 2007), and maltreatment that is associated with an increased risk of teenage delinquency (Aslund et al., 2011). Additionally, it is important to study females with CD because of the sex differences in neural structures associated with antisocial and aggressive behaviours (Gur, Gunning-Dixon, Bilker, & Gur, 2002), in the rate of maturation (Perrin et al., 2009), and in the neural mechanisms underlying the processing of emotions, importantly in the perception of emotion and down-regulation of negative emotions (Whittle, Yücel, Yap, & Allen, 2011).

Presently, however, there are few studies of the neuroanatomical correlates of CD among females. In a small sample of adolescent girls, those with CD, relative to healthy girls, showed reduced GMV in the bilateral anterior insula and right striatum. As is typical of children and adolescents with CD, the girls with CD presented high rates of comorbid disorders, some of which were associated with specific abnormalities (Fairchild et al., 2013). Among female juvenile delinquents, psychopathy scores were negatively correlated with GMV in limbic and paralimbic areas, including the orbital frontal cortex (OFC), parahippocampal cortex, temporal poles, and left hippocampus (Cope, Ermer, Nyalakanti, Calhoun, & Kiehl, 2014). By contrast, a recent study did not observe differences in GMV between girls with and without CD but did detect a quadratic negative association between the number of CD symptoms and GMV in the left superior temporal sulcus with a trend on the right (Michalska, Decety, Zeffiro, & Lahey, 2015)*.* Another recent study reported that adolescent girls with severe substance misuse and conduct problems, relative to healthy girls, displayed less grey matter volume in right dorsolateral prefrontal cortex, left ventrolateral prefrontal cortex, medial orbitofrontal cortex, anterior cingulate, bilateral somatosensory cortex, left supramarginal gyrus, and bilateral angular gyrus (Dalwani et al., 2015). However the study selected cases with both conduct and substance misuse problems and only 14 out of 22 of these females met criteria for CD.

Samples that included both girls and boys with CD observed GMV reductions in the amygdala and striatum using Freesurfer (Wallace et al., 2014) and reductions in cortical thickness in temporal and parietal regions (Hyatt, Haney-Caron, & Stevens, 2012; Wallace et al., 2014). Thus, there is limited evidence that girls with CD display alterations in GMV in limbic and paralimbic areas. Further, it is not known whether young women who had presented CD in adolescence would show alterations of brain structure as compared to women who had never presented CD or, importantly, whether observed abnormalities would be associated with the comorbid disorders and experiences of trauma that are common among children and adolescents with CD.

Typically, significant proportions of adolescents with CD and adults with ASPD present comorbid externalizing disorders, specifically alcohol misuse (Alegria, Blanco, & Petry, 2013) and drug misuse (Nock, Kazdin, Hiripi, & Kessler, 2006), internalizing disorders, specifically anxiety disorders (Angold, Costello, & Erkanli, 1999; Goodwin & Hamilton, 2003), and depression disorders (Greene, 2002), and histories of physical and sexual abuse (Afifi, Boman, Fleisher, & Sareen, 2009). Each of these conditions has been associated with alterations of GMV (Fein et al., 2002; Liu et al., 2014; Niciu & Mason, 2014; Rando, Tuit, Hannestad, Guarnaccia, & Sinha, 2012; Schienle, Ebner, & Schäfer, 2011; Shang et al., 2014; Walsh et al., 2014).

Consequently, knowledge of the neural basis of CD will be furthered by identifying differences in GMV associated with CD after taking account of comorbid disorders and childhood maltreatment.

## The present study

The present study aimed to determine whether young women who had presented CD prior to age 15 (CDW) present alterations in GMV as compared to healthy women (HW). Using voxel-based morphometry, exploratory whole-brain analyses were conducted and five regions of interest (ROI) examined. Given the few studies of brain morphology among antisocial females, the ROIs were selected based on evidence from both females and males. We hypothesized that CDW, relative to HW, would show bilateral reductions of GMV in the (1) anterior insula, based on previous findings among both females (Fairchild et al., 2013) and males with CD (Fairchild et al., 2011; Sterzer et al., 2007); (2) amygdala, based on evidence in male adolescents with CD (Fairchild et al., 2011; Sterzer et al., 2007); (3) hippocampus, based on findings in females with psychopathic traits (Cope, Ermer, Nyalakanti, et al., 2014) and male offenders with ASPD (Laakso et al., 2001); (4) OFC, based on findings in female delinquents (Cope et al., 2014) , boys with CD (Huebner et al., 2008), and adults with CD/ ASPD (Raine, Yang, Narr, & Toga, 2011); and (5) anterior cingulate cortex (ACC), based on studies of boys with CD (De Brito et al., 2009), adult males with CD/ASPD (Kumari et al., 2014). In order to disentangle neural correlates of CD from those of past comorbid disorders and maltreatment, group comparisons were computed, controlling for past alcohol and drug dependence, anxiety and depression disorders, and maltreatment (physical and sexual abuse). In a second step, to investigate the effect of current measure of comorbid psychopathology, group comparisons were rerun, controlling for current use of alcohol and drugs and anxiety and depression symptoms.

## Method

### Participants

The sample included 31 women diagnosed with CD prior to age 15. Twenty-five of these women consulted a clinic for substance misuse in adolescence (Hodgins et al., 2007), and six women were sisters of other attendees of the clinic who themselves did not participate in the present study; thus, none of the CDW were related to each other. Twenty-five of the CDW were first assessed (Hodgins et al., 2007) in midadolescence when those 18 years or younger completed the Schedule for Affective Disorders and Schizophrenia for School-Age Children (Kaufman et al., 1997) and those 18 or older the Structured Clinical Interview for DSM-IV (SCID I, SCID II; First, Gibbon, Williams, & LS, 1990; First, Spitzer, Miriam, & Williams, 2002), and reassessed 6, 12, and 60 months later (Hodgins, Lövenhag, Rehn, & Nilsson, 2014; Hodgins, Oliver, Tengström, & Larsson, 2010). Six of the CDW were assessed at the 60 month follow-up and approximately 18 months later when the scans were done. Before the scan, the HW completed the SCID I and II, and all of the CDW completed the SCID I. HW included 25 females with no history of CD, or criminal behavior, no current or past Axis I or II disorders other than two cases of past alcohol abuse. HW were recruited by announcements placed on company bulletin boards and on the Internet. The sample provided adequate power (83%) to detect a large difference (Cohen’s *d* = 0.8) between the CDW (*n* = 31) and HW (*n* = 25) using *t* tests (Cohen, 1988; Faul, Erdfelder, Lang, & Buchner, 2007). No participant had neurological illness, loss of consciousness for more than 30 minutes, or any other contraindication for a MRI brain scan.

### Procedure

CD women were initially contacted by letter, and then by telephone, requesting their participation in brain imaging study. If they agreed, an appointment for an interview and scan was scheduled. HW were initially screened on the telephone. If they met eligibility criteria, an interview and scan was scheduled.

All participants were asked to refrain from alcohol and drug use for 3 days prior to scanning. On arriving for the interview and scan, the study was explained again to participants and written consent obtained. Using a breath analyzer and saliva sample, participants were screened for recent use of alcohol and seven classes of illegal drugs. None tested positive. HW completed an interview to diagnose mental disorders, questionnaires, and an IQ test. CDW completed only the diagnostic interview as other information was available from past assessments. Additionally, at the time of the scan all participants completed questionnaires to assess current alcohol and drug use and anxiety and depression symptoms.

### Measures

#### Handedness

Handedness was assessed using the Edinburgh Handedness Inventory (Oldfield, 1971).

#### IQ

IQ was estimated using the vocabulary and block design subtests of the Wechsler Intelligence Scale. CDW who were ages 16 or younger at the first assessment completed the Wechsler Intelligence Scale for Children, Third Edition (Wechsler, 1991), and all other participants completed the Wechsler Adult Intelligence Scale, Revised (Wechsler, 1997).

#### Comorbid disorders

Past and current (past 6 months) externalizing disorders were defined as presence of diagnoses of alcohol and/or drug dependence at any of the past assessments. Past and current internalizing disorders were defined to include depressive disorders (major depressive disorder, dysthymia, depressive disorder not-otherwise-specified or substance-induced mood disorder) and anxiety disorders (agoraphobia, generalized anxiety disorder, anxiety disorder not-otherwise-specified, obsessive compulsive disorder, panic disorder, posttraumatic stress disorder, social phobia, specific phobia or substance-induced anxiety disorder).

#### Psychopathy affective facet

Of the CDW, 25 were assessed using the Psychopathy Checklist: Youth Version (PCL:YV) (Forth, Kosson, & Hare, 2003) in midadolescence, and 6 of the CDW and all the HW were assessed using the Psychopathy Checklist Screening Version (PCL:SV) (Hart, Cox, & Hare, 1995), at the time of the scan. As PCL:YV Factor 2 scores vary from 0 to 8 and PCL:SV Factor 2 scores from 0 to 6, each score was divided by the total possible score.

#### Physical abuse

The revised Conflict Tactics Scales (Straus, Boney-McCoy, & Sugarman, 1996) were used to assess physical abuse by parents, defined as a report by the participant that she had been hit with a fist or kicked hard, hit on a part of the body other than the bottom with a hard object, thrown or knocked down, grabbed around the neck and choked, beaten up, hit repeatedly very hard, burned, or threatened with a gun or knife.

#### Sexual abuse

Sexual abuse was assessed using the Sexual and Physical Abuse Questionnaire (Kooiman, Ouwehand, & ter Kuile, 2002), MacArthur Community Violence Instrument (Steadman et al., 1998), and the Sexual Experience Survey (Koss et al., 2006)). Sexual abuse was coded present if any of the following were reported: forced to have sex against her will by a person in authority, by offering alcohol or drugs, or by physical violence.

#### Current alcohol use

Current (past 6 month) alcohol use was assessed by self-report using the Alcohol Use Disorders Identification Test (AUDIT) (Saunders, Aasland, Babor, de la Fuente, & Grant, 1993).

#### Current drug use

Current (past 6 month) drug use was assessed using by self-report using the Drug Use Disorders Identification Test (DUDIT) (Berman, Bergman, Palmstierna, & Schlyter, 2003).

#### Current anxiety symptoms

Current anxiety symptoms (past 7 days) were self-reported using the Beck Anxiety Inventory (BAI) (Beck, Epstein, Brown, & Steer, 1988).

#### Current depression symptoms

Current depression symptoms (past 7 days) were self-reported using the Beck Depression Inventory (BDI) (Beck, Ward, Mendelson, Mock, & Erbaugh, 1961).

#### Current psychosocial functioning

Full-time activity (work, education, job training) during the past 2 years was self-reported, as was having a child. Aggressive behavior in the last 6 months was assessed using the MacArthur Community Violence Instrument (Steadman et al., 1998).

### MRI data acquisition

MRI scans were acquired with a 3-Tesla MRI-scanner (MR750 GE Healthcare, Milwaukee, Wisconsin) using an eight channel phased array receiving coil, at the Karolinska University Hospital. Axial isotropic 3-D T1 images were acquired with 176 slices of 1-mm thickness, voxel size = 1 × 1 × 1 mm, FOV = 24.0, Flip angle = 12 degrees, matrix = 240 × 240, TR = 7.9 ms, TI = 450 ms, and TE = 3.1 ms. Total scan time to acquire T1 images was 6 minutes, 8 seconds. Images were inspected for clinically relevant abnormalities by a radiologist.

### Image processing

Image processing was performed in Statistical Parametric Mapping Version 8 software (SPM8; Wellcome Trust Centre for Neuroimaging, University College, London), implemented in MATLAB R2010a (MathWorks Inc., Natick, MA, USA). MRI images were displayed in SPM8 to visually inspect for gross anatomical abnormalities and scanner artifacts for each subject. Images were manually reoriented and realigned to the anterior commissure. The new segment feature in SPM8 was used to segment the images into gray matter, white matter, and cerebrospinal fluid. The DARTEL (diffeomorphic anatomical registration through exponentiated lie algebra) toolbox (Ashburner, 2007) in SPM8 was used to create an average template by using gray matter, white matter, and cerebrospinal fluid segmentations of all 56 subjects. Gray matter segmentations for each participant were warped to the template generated by DARTEL, interpolated to a 1.5-mm isotropic voxel resolution and transformed to Montreal Neurological Institute (MNI) space. Normalized gray matter images were then Jacobian scaled modulated to preserve the total amount of gray matter in each voxel.

These Jacobian-modulated images were smoothed using an 8-mm full-width at half-maximum Gaussian kernel. Final output images were smoothed, modulated, normalized GMV of 1.5-mm^3^ voxel size.

### Statistical analyses

Independent sample *t* tests and Fischer’s exact tests were conducted, using the Statistical Package for Social Sciences (SPSS, Version 21) to compare groups on sociodemographic and clinical characteristics. Differences in GMV between women with CD and HW were examined using a general linear model. In order to detect group differences, statistical parametric maps were computed on a voxel-by-voxel basis for GMV, and independent two-sample *t* tests were used. In order to avoid possible edge effects between different tissue types, we excluded all voxels with gray matter values less than 0.1 (absolute threshold masking). Total intracranial volume (TIV) was calculated as the sum of gray matter, white matter, and cerebrospinal fluid volumes, as estimated by the MATLAB get_totals script (http://www.cs.ucl.ac.uk/staff/g.ridgway/vbm/get_totals.m). First, we conducted exploratory between-group GMV whole-brain comparisons controlling for total intracranial volume (TIV). The uncorrected statistical threshold for voxels in the exploratory whole-brain analysis was set at *p* < .001. The significance threshold for whole-brain analyses was set at *p* < .05, using family-wise error (FWE) correction for multiple testing at voxel/cluster level. In order to correct for the nonisotropic smoothness of the data, nonstationary cluster correction was applied (Hayasaka, Phan, Liberzon, Worsley, & Nichols, 2004). Second, we used the small volume correction (SVC) approach to restrict our analysis to the predefined ROIs. ROIs were created for each hemisphere, using the Mars bar (MRC Cognition and Brain Sciences Unit, Cambridge, UK) and atlas for automated anatomical labeling (Tzourio-Mazoyer et al., 2002). The ROI for ACC is limited by the paracingulate sulcus rostrally and the white matter of the corpus callosum caudally. The hippocampus included the dentate gyrus, the uncus, and the hippocampus proper. We combined the mask of superior, medial, middle and inferior subregions of the OFC to get one mask for OFC. The anterior insula was defined by restricting the insula mask to the region anterior to the anterior commissure plane (i.e., *y* > 0). Clusters were reported as significant if they survived FWE correction for multiple comparisons, significance level of *p* < .05, within the ROIs.

Two sets of analyses were conducted in attempt to disentangle neural correlates of CD and those of comorbid disorders and maltreatment. Step 1 aimed to determine whether observed group differences would remain after comparisons were adjusted for past comorbid disorders and maltreatment. Four models were computed. All models adjusted for TIV, age, and IQ. In addition, Model 1 adjusted for past alcohol and drug dependence, Model 2 adjusted for past anxiety and depression disorders, Model 3 for history of physical and sexual abuse, and Model 4 for all comorbid disorders and maltreatment. Since few of the participants presented comorbid disorders in adulthood when undergoing the brain scan, Step 2 aimed to determine whether differences between those with prior CD and HW would remain after adjusting for current alcohol and drug use, anxiety and depression symptoms, and all continuous measures. Three models were computed, again adjusting for TIV, age, and IQ. Model 1 adjusted for current alcohol and drug use, Model 2 for current anxiety and depression symptoms, and Model 3 for all current measures. PCL Facet 2 scores were low. However, to determine their association with GMV, regression analyses were computed to estimate the association with whole brain and ROIs after adjusting for TIV, age, and IQ.

### Ethics approval

The current study, and all previous waves of data collection, was approved by the Regional Ethical Review Board in Stockholm.

## Results

### Sociodemographic and clinical characteristics of participants

As presented in Table 1, CDW and HW were similar as to age and handedness (*p* > .05), and differed as to IQ and the proportions with high school diplomas and PCL Facet 2 scores. As is typical, proportionately more of the CDW than the HW acquired past diagnoses of alcohol dependence, drug dependence, anxiety disorders, depression disorders and reported physical and sexual abuse. At an average age of 24 years, approximately 10% of the CDW met criteria for ASPD, few presented substance use disorders, one third presented an anxiety disorder, and 13% had depression disorder. None of the participant presented current or past histories of psychosis, bipolar disorder, autism, or Cluster A or C personality disorders. At the time of the scan, the CDW were similar to HW as to alcohol use but presented higher drug use and anxiety and depression symptoms relative to HW. More than half of the CDW had not been employed in the previous 2 years, their scores for aggressive behavior were high, and almost half had at least one child (10 years earlier than the average age at first birth of women in Stockholm).

**Table 1:** Comparisons of women with conduct disorder prior to age 15 and healthy women

	Conduct disorder (*n* = 31)	Healthy (*n* = 25)	Statistics
Mean (*SD*) age (years)	24.1 (2.7)	22.7 (3.3)	*t*(54) = 1.80, *p* = .08
Mean (*SD*) score handedness	87.6 (33.6)	92.0 (10.3)	*t*(54) = 1.19, *p* = .282
Mean (*SD*) number of conduct disorder symptoms prior to age 15	5.8 (2.8)	0	
Mean (*SD*) PCL Facet 2 scores	0.33 (0.27)	0.19 (0.05)	*t*(54) = 5.516, *p* < .001
Mean (*SD*) number of aggressive conduct disorder symptoms	1.5 (1.6)	0	
Mean (*SD*) IQ scores	16.5 (3.9)	19.3 (2.6)	*t*(53) = 3.16, *p* = .003
% Completed high school	45	80	FET *p* = .008
Past comorbid disorders			
% Alcohol dependence	39	0	FET *p* < .001
% Drug dependence	42	0	FET *p* < .001
% Anxiety disorder	80	0	FET *p* < .001
% Depression disorder	67	0	FET *p* < .001
% Attention-deficit/hyperactive disorder	9.7	0	FET *p* = .167
Maltreatment			
% Physical abuse by parents	38	0	FET *p* < .001
% Sexual abuse	61	0	FET *p* < .001
At the time of the scan			
Participants taking medication^A^	3	0	
% Not employed in past 2 years	58.6	0	FET, *p* = .001
% Alcohol dependence	3.2	0	FET *p* = .554
% Drug dependence	6.5	0	FET *p* = .302
% Anxiety disorder	32	0	FET *p* = .001
% Depression disorder	13	0	FET *p* = .08
% Antisocial personality disorder	9.7	0	FET *p* = .162
Mean (*SD*) AUDIT scores	6.4 (4.06)	4.8 (3.3)	*t*(54) = 1.61, *p* = .107
Mean (*SD*) DUDIT scores	4.5 (8.41)	0.24 (0.83)	*t*(54) = 2.76, *p* = .016
Mean (*SD*) BAI scores	11.41(10.1)	4.96 (4.5)	*t*(54) = 3.17, *p* = .005
Mean (*SD*) BDI scores	13.77(14.1)	3.26 (4.5)	*t*(54) = 3.90, *p* = .001
% with children	48	12	FET *p* = .004
Mean (*SD*) score aggressive behavior last 6 months	0.87 (1.60)	0.12 (.439)	*t*(54) = 2.48, *p* = .018

*Note.* One participant was taking methylphenidate, two were taking fluoxetine

FET = Fischer Exact Test

AUDIT = Alcohol Use Disorder Identification Test

DUDIT = Drug Use Disorder Identification Test

BAI = Beck Anxiety Inventory

BDI = Beck Depression Inventory

PCL = Psychopathy Checklist: Youth Version (*n* = 25) and Psychopathy Checklist: Screening Version affect Facet 2 scores (*n* = 31). Scores are presented as a fraction of total possible score

### Global volume

As presented in Table 2, relative to HW, CDW were characterized by significantly less TIV, total tissue volume, and total GMV. The group differences in total GMV were no longer significant after adjusting for past alcohol and drug dependence, past anxiety and depression, and current anxiety and depression symptoms but remained significant after adjusting for physical and sexual abuse and current alcohol and drug use. Group differences in white matter and cerebrospinal fluid volume were not statistically significant.

**Table 2:** Comparisons of global volume measures (cm^3^) of women with conduct disorder prior to age 15 and healthy women

Global volume measures	Women with conduct disorder	Healthy women	Statistics *t*(*df*) *p* value
Mean (*SD*) total intracranial volume	1410.37 (80.57)	1461.10 (71.00)	2.46(54) *p* = .017
Mean (*SD*) total tissue volume	1132.72 (69.23)	1175.62 (56.16)	2.50(54) *p* = .015
Mean (*SD*) total gray matter volume	674.13 (40.33)	701.69 (32.01)	2.78(54) *p* = .007^a^
Mean (*SD*) total white matter volume	458.60 (30.89)	473.92 (26.60)	1.96(54) *p* = .06
Mean (*SD*) total cerebral spinal fluid volume	277.64 (15.39)	285.47 (18.93)	1.70(54) *p* = .10

^a^ Adjusted for past alcohol and drug dependence, *F* = 0.871, *df* = 1, *p* = .355; adjusted for past anxiety and depression disorders, *F* = 1.523, *df* = 1, *p* = .223; adjusted for physical and sexual abuse, *F* = 4.211 *df* = 1, *p* = 0.046; adjusted for all past co-variates, *F* = 1.876, *df* = 1, *p* = .177; adjusted for current alcohol and drug use, *F* = 4.529, *df* = 1, *p* = 0.38; adjusted for current anxiety and depression, *F* = 2.769, *df* = 1, *p* = .102; adjusted for all current, *F* = 3.043, *df* = 1, *p* = .089

### Whole brain analyses

**Fig. 1:**
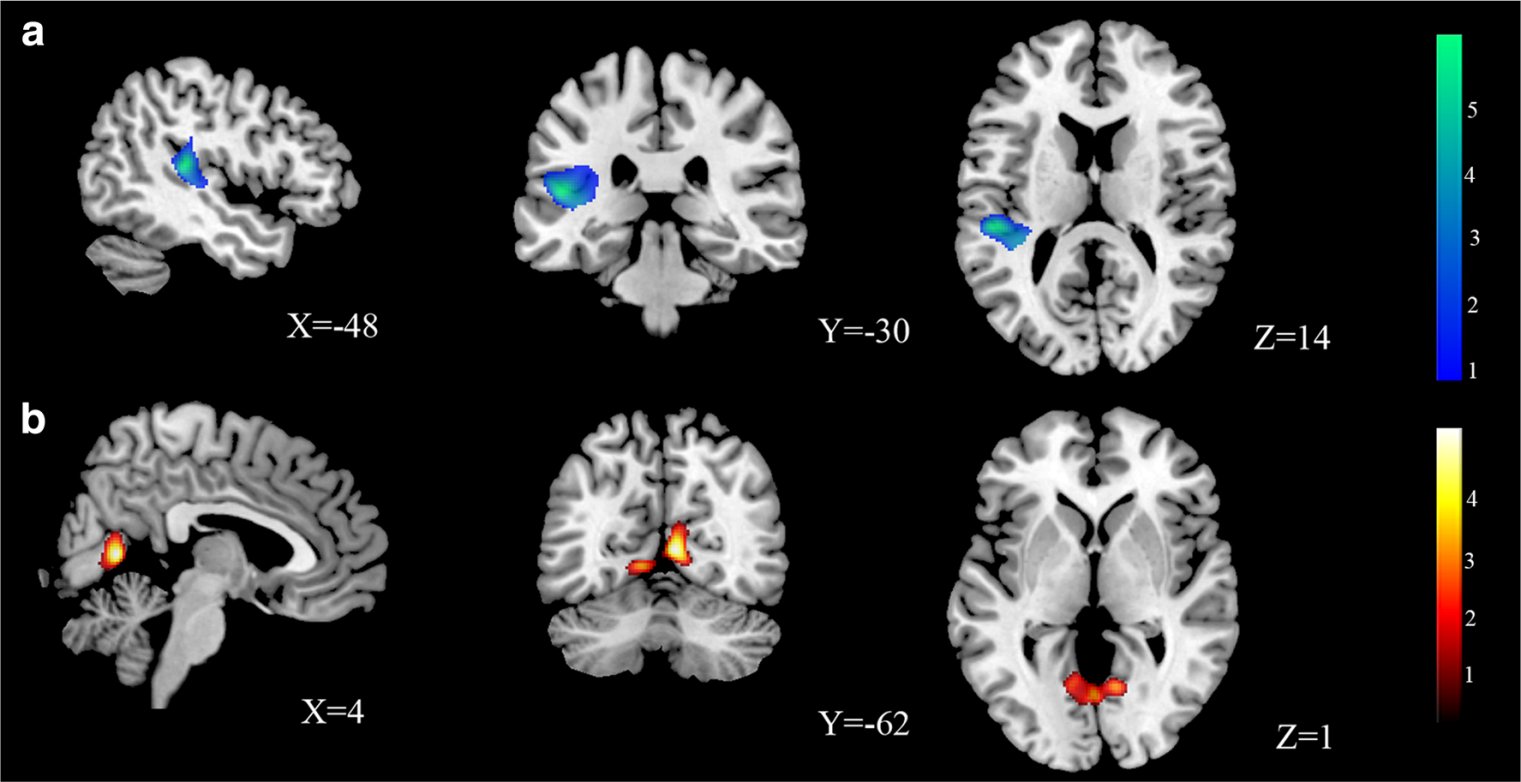
Whole-brain voxel-based morphometry analysis of gray matter volume where significant differences were observed after correcting for multiple comparisons. **a** Increased gray matter volume (pFWE < .05) in left superior temporal gyrus among women with conduct disorder compared to the healthy women. **b** Reduced gray matter volume (pFWE < .05) in lingual gyrus among women with conduct disorder compared to healthy women. *Color bar* represents *t* scores. (Color figure online)

After adjusting for TIV, the CDW, relative to HW, showed significantly increased GMV in a large cluster including the left superior temporal gyrus (STG) extending upto posterior insula/parietal operculum regions (FWE corrected *p* = .040, at the cluster level, FWE corrected *p* = .004, at the peak level, *T* = 6.01, *Z* = 5.23, extent = 1,274 voxels), and reduced GMV in a large cluster located in the lingual gyrus extending up to the fusiform gyrus (FWE corrected *p* = .028, at the cluster level, *T* = 5.05, *Z* = 4.54, extent = 989 voxels; see Fig. 1). The coordinates of these areas are shown in Table 3.

**Table 3:** Differences in regional gray matter volumes of women with conduct disorder prior to age 15 and healthy women after adjusting for past comorbid disorders and maltreatment

Whole brain group differences													
						Adjusted for total intracranial volume, age, IQ, and additional covariates							
Brain regions	MNI coordinates			Total intracranial volume		Alcohol and drug dependence		Anxiety and depression disorders		Physical and sexual abuse		All covariates	
	*X*	*Y*	*Z*	Cluster size	*t* score	Cluster size	*t* score	Clustersize	*t* score	Cluster size	*t* score	Cluster size	*t* score
**Conduct disorder > Healthy**													
Left superior temporal gyrus	−48	−30	12	1274	**6.01** ^*^	783	5.02^**^	708	**5.49** ^*^	27	3.61^**^	250	4.92^**^
	−50	−27	17										
**Conduct disorder < Healthy**													
Lingual gyrus	4	−63	6	989	**5.05** ^*^	458	4.31^**^			390	4.20^**^		
	−10	−62	−3										
	0	−75	−7										
	9	−65	2										
**Regions of interest: Group differences**													
**Conduct disorder < Healthy**													
Left hippocampus	−18	−36	0	30	**3.63** ^***^								
Left anterior cingulate cortex	0	27	26	168	**3.89** ^***^					224	**3.79** ^***^		
	−6	33	20										

Boldface t score indicate that the results were significant ﻿after family wise error (FWE) correction

Empty cells indicate that the group difference disappeared after adjustment for the covariate

*p < .05, with family-wise error (FWE) correction for multiple comparisons

**p < .001, uncorrected

***p < .05, with family-wise error (FWE) correction for multiple comparisons after small volume correction

### Regions of interest

Region of interest analyses showed that after small volume correction, GMV of the left hippocampus (FWE corrected *p* = .048, at the cluster level, *T* = 3.60, *Z* = 3.39, extent = 30 voxels) and left ACC (FWE corrected *p* = .039, at the cluster level, *T* = 3.89, *Z* = 3.63, extent = 168 voxels) were significantly reduced in the CDW relative to HW (see Table 3). No group differences were detected in the amygdala, right hippocampus, anterior insula, and OFC.

### Adjusting for past comorbid disorders and trauma

Table 3 presents results of comparisons of GMV of CDW and HW initially adjusting only for TIV, and then for TIV, age, IQ, past comorbid disorders and maltreatment. The increased GMV in STG, posterior insula/parietal operculum shown by the CDW relative to HW remained significant after adjusting for anxiety and depression disorders (FWE corrected *p* = .026, at the voxel level, *T* = 5.49, *Z* = 4.87). The group difference was not significant after FWE correction and reduced to trend in model 1 (pFWE = .097, *p* < .001, uncorrected, *T* = 5.02, *Z* = 4.48) that adjusted for alcohol and drug dependence, in Model 3 (pFWE = .99, *p* < .001, uncorrected, *T* = 3.61, *Z* = 3.37) that adjusted for physical and sexual abuse, and in Model 4 (pFWE = .164, *p* < .001, uncorrected, *T* = 4.92, *Z* = 4.34) that adjusted for all comorbid disorders and maltreatment.

The group difference in GMV of the lingual gyrus was not significant after FWE correction and reduced to a trend after adjusting for past alcohol and drug dependence (pFWE = .33, *p* < .001, uncorrected, *T* = 4.31, *Z* = 3.95), and physical and sexual abuse (pFWE = .495, *p* < .001, uncorrected, *T* = 4.20, *Z* = 3.85). The group difference disappeared in Model 2 that adjusted for past anxiety and depression disorders and Model 4 that adjusted for all disorders and maltreatment.

The GMV reduction in the left hippocampus shown by the CDW relative to HW was not significant in any of the adjusted models. The GMV reduction in the left ACC that was observed among the CDW was no longer significant in Model 1 that adjusted for alcohol and drug dependence and Model 2 that adjusted for anxiety and depression but remained significant in the Model 3 (FWE corrected *p* = .039, at the cluster level, *T* = 3.79, *Z* = 3.52) that adjusted for physical and sexual abuse.

### Adjusting for current substance use and anxiety and depression symptoms

Table 4 presents results of GMV comparisons of CDW and HW adjusting for TIV, age, IQ, and scores for current alcohol use, drug use, anxiety symptoms, and depression symptoms. The increased GMV in STG, posterior insula/parietal operculum shown by the CDW relative to HW was not significant after FWE correction and reduced to a trend after adjusting for alcohol and drug use (pFWE = .29, *p* < .001, uncorrected, *T* = 4.58, *Z* = 4.15), after adjusting for anxiety and depression symptoms (pFWE = .53, *p* < .001, uncorrected, *T* = 4.29, *Z* = 3.93), and after adjusting for both substance use and symptoms (pFWE = .614, *p* < .001, uncorrected, T = 4.23, *Z* = 3.86). The group difference in GMV of the lingual gyrus was also not significant after FWE correction and reduced to a trend after adjusting for current alcohol and drug use (pFWE = .48, *p* < .001, uncorrected, *T* = 3.85, *Z* = 3.57), anxiety and depression symptoms (pFWE = .98, *p* < .001, uncorrected, *T* = 3.62, *Z* = 3.39), and both substance use and symptoms (pFWE = 0.97, *p* < .001, uncorrected, *T* = 3.60, *Z* = 3.36). The observed GMV reduction in the left hippocampus and left ACC shown by the CDW relative to HW was not significant in the models adjusted for current substance misuse and/or symptoms.

**Table 4:** Differences in regional gray matter volumes of women with conduct disorder prior to age 15 and healthy women after adjusting for current alcohol and drug use and symptoms of anxiety and depression

	Whole brain group differences										
						Adjusted for total intracranial volume, age, IQ and additional covariates					
Brain regions	MNI coordinates			Total intracranial volume		AUDIT and DUDIT scores		BAI and BDI scores		All	
	*X*	*Y*	*Z*	Cluster size	*t* score	Cluster size	*t* score	Cluster size	*t* score	Cluster size	*t* score
**Conduct disorder > Healthy**											
Left superior temporal gyrus	−48	−30	12	1274	**6.01** ^*^	395	4.58**	176	4.29**	108	4.23**
	−50	−27	17								
**Conduct disorder < Healthy**											
Lingual gyrus	4	−63	6	989	**5.05** ^*^	342	3.85**				
	−10	−62	−3					51	3.62**	41	3.60**
	0	−75	−7								
	9	−65	2					30	3.44**		
Regions of interest: Group differences											
**Conduct disorder < Healthy**											
Left hippocampus	−18	−36	0	30	3.63***						
Left anterior cingulate cortex	0	27	26	168	3.89***						
	−6	33	20								

Boldface t score indicate that the results were significant ﻿after family wise error (FWE) correction

Empty cells indicate that the group difference disappeared after adjustment for the covariate

*p < .05, with family-wise error (FWE) correction for multiple comparisons

**p < .001, uncorrected

***p < .05, with family-wise error (FWE) correction for multiple comparisons after small volume correction

### Post hoc analyses

Since only three of the CDW met diagnostic criteria for ASPD, group comparisons were rerun excluding these participants. All results were similar except that there was no difference in GMV of the hippocampus.

After adjusting for TIV, age, and IQ, PCL affective Facet 2 scores were not associated with GMV in any brain region in whole-brain analyses or in the ROIs.

**Fig. 2:**
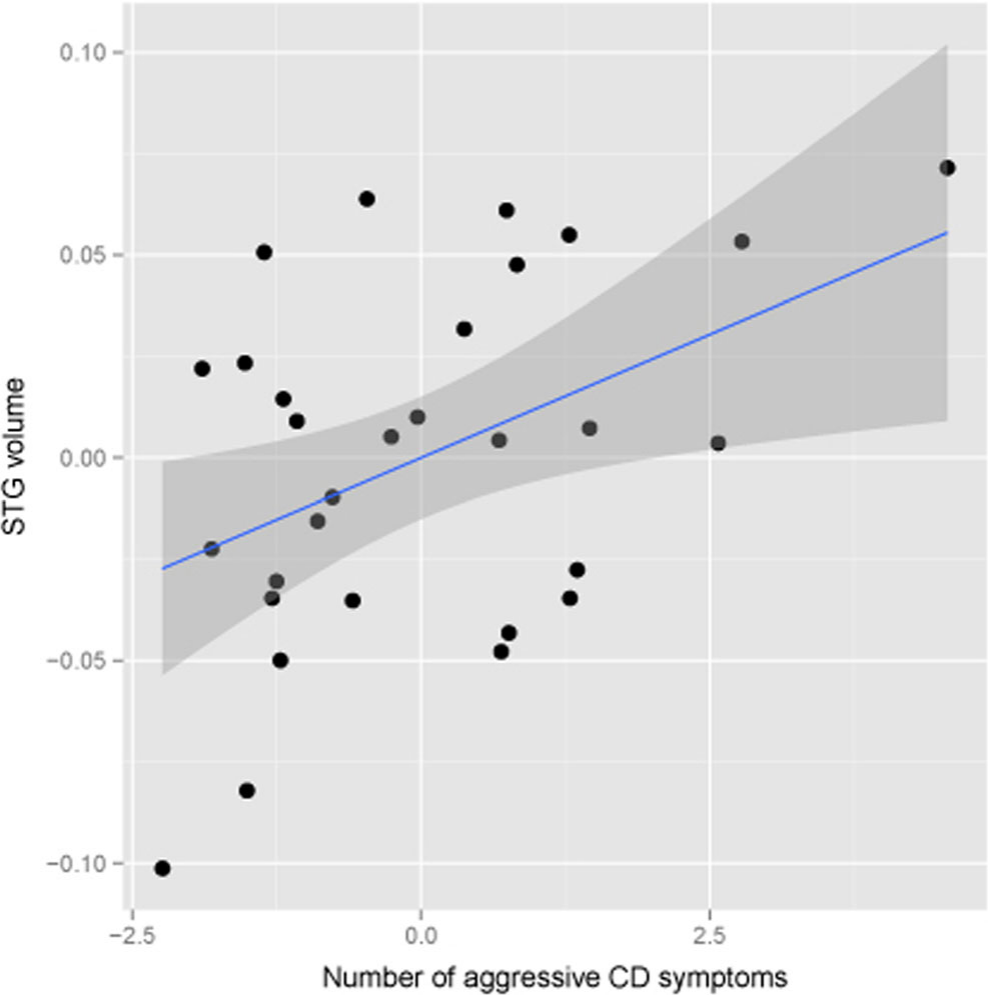
Scatter plots show partial correlations of number of aggressive conduct disorder symptoms and gray matter volume at the local maxima of left superior temporal gyrus, after adjusting for total intracranial volume, age, and IQ. Residual aggressive symptoms and residual STG volume (after correcting for age, IQ, and total intracranial volume) are used in the scatterplot to show linear relationship between number of aggressive conduct disorder symptoms and gray matter volume of left superior temporal gyrus. The *blue line* is slope of correlation and *gray area* depicts 95% confidence interval. (Color figure online)

The group difference in GMV of the STG was reduced to a trend after adjusting for comorbid disorders and maltreatment. Despite this reduction in statistical significance, we examined the association with the number of CD symptoms. For the CDW, GMV of STG cluster was extracted by volume-of-interest analysis (first eigenvariate), and correlations with numbers of nonaggressive and aggressive CD symptoms were calculated. As shown in Fig. 2, after controlling for TIV, age, and IQ, GMV in the STG was positively, significantly associated with the number of aggressive CD symptoms (part *r* = .42, *p* = .021) and was not associated with number of nonaggressive symptoms.

## Discussion

Young women who had presented CD before age 15 were characterized by wide-spread abnormalities of GMV relative to HW. All of these GMV abnormalities were observed at an average age of 24 years, when few of these CDW met the diagnostic criteria for ASPD, substance dependence and anxiety and depression disorders. Typical of children/adolescents with CD, the majority of the CDW had acquired past diagnoses of anxiety and depression disorders, alcohol and drug dependence and had experienced maltreatment. The observed abnormalities of GMV were associated with past comorbid disorders and maltreatment and with current substance misuse and symptoms of anxiety and depression in women with prior CD. In whole-brain analyses, relative to HW, women with CD displayed increased GMV in the STG and posterior insula parietal operculum regions and decreased GMV in the lingual gyrus. While comparing groups in a priori ROIs, reduced GMV in left hippocampus and left ACC were observed in women with CD, relative to HW. Importantly, after adjusting group comparisons for past comorbid disorders and maltreatment and current substance use and symptoms of anxiety and depression, none of the observed group difference remained significant after FWE correction.

The CDW showed increased GMV in the left STG extending up to the posterior insula/parietal operculum regions that remained significant after taking account of TIV, age, IQ, and past internalizing disorders. The group difference was not significant after FWE correction when adjusted for current anxiety and depression symptoms. Increased GMV in STG is reported in children and adolescents with generalized anxiety disorder, in comparison to healthy control subjects (De Bellis, Keshavan, Shifflett, et al., 2002). These results are consistent with evidence suggesting functional hyperactivity in bilateral STG during processing of threatening words, in adults with anxiety disorders, relative to healthy adults (Zhao et al., 2014). The reason why current anxiety and depression symptoms were associated with STG GMV and not past anxiety and depression disorders remains unknown.

The group difference in STG was also associated with past diagnoses of alcohol and drug dependence or current alcohol and drug use. The result is consistent with findings from a recent study of male adolescents with substance use disorders and conduct problems, which reported a positive association between the number of conduct disorder symptoms and cortical thickness in STG (Chumachenko et al., 2015).

The group difference in GMV in the STG was reduced substantially when controlling for childhood maltreatment, suggesting that observed GMV abnormalities of STG among women with CD may be associated with maltreatment. This finding is in line with a study that found maltreatment was associated with increased GMV in the STG (De Bellis, Keshavan, Frustaci, et al., 2002). Maltreatment is a trauma that activates the body’s biological stress response systems (De Bellis & Zisk, 2014). Elevated levels of stress hormones and neurotransmitters may lead to adverse brain development through delays in myelination and alterations in developmentally appropriate pruning (De Bellis & Zisk, 2014; Lauder, 1988; Todd, 1992). Subconscious recall of trauma may lead to hypervigilance and emotional hyperreactivity (De Bellis et al., 2002) that is associated with reactive aggressive behavior typical of individuals with CD.

The STG plays an important role in social cognition, particularly in initial stages of evaluating the intentions of others during analysis of eye-gaze direction, facial expressions, and body movements (Allison, Puce, & McCarthy, 2000). Girls with CD show marked impairments in facial emotion recognition that are most severe for anger and disgust (Fairchild, Stobbe, van Goozen, Calder, & Goodyer, 2010). Children with CD view neutral faces as threatening (Dadds et al., 2006), which is associated with an increase in the likelihood of reactive aggressive behavior (Crick & Dodge, 1996). Moreover, the STG works in concert with other frontal and subcortical regions to process information involved in complex social cognition and emotion.

Adult males with prior CD, relative to healthy males, were reported to show increased GMV in the STG in a large cluster including the uncus and the superior temporal cortex (Schiffer et al., 2013). Among children with conduct problems, relative to healthy children, both increased (De Brito et al., 2009) and decreased (Huebner et al., 2008) GMV in temporal lobes have been reported. Results of the present study suggest that these contradictory results may be due, at least in part, to sample differences in the proportions of participants who have been maltreated or who have begun to misuse substances. Given the elevated prevalence of maltreatment and substance misuse among children and adolescents with CD, findings from the present study highlight the importance of taking account of these comorbid disorders and history of maltreatment when attempting to identify the neural correlates of CD. Further, they highlight the importance of conducting prospective studies to determine whether maltreatment leads to abnormality of the STG that in turn increases the likelihood of CD and the typically associated substance use and internalizing disorders.

The CDW, relative to HW, displayed decreased GMV in the lingual gyrus, bilaterally. The group difference in GMV of the lingual gyrus, bilaterally, did not survive FWE adjustment for current anxiety and depression, past and current substance misuse, and maltreatment. Perhaps, importantly, the group difference disappeared when adjusted for past anxiety and depression disorder. Internalizing disorders afflict as many as 50% of children with CD and adults with ASPD who show high levels of aggressive behavior (Polier, Vloet, Herpertz-Dahlmann, Laurens, & Hodgins, 2012) and comorbidity between internalizing problems and antisocial behaviors may involve common risk factors (Wolff & Ollendick, 2006). Reduced GMV of the lingual gyrus has been observed among adults with posttraumatic stress disorder and among those with depression disorders (Nardo et al., 2013). In a recent study, reduced cortical thickness is observed in patients with depression disorders in comparison to healthy controls (Na et al., 2016). Among teenage boys, those with serious conduct and substance problems relative to healthy boys, showed reduced GMV in the right lingual gyrus, with no adjustment for comorbid anxiety or maltreatment (Dalwani et al., 2011). It could be that reduced GMV in the lingual gyrus may add to an abnormality of the STG to further impair comprehension of emotions in the faces of others and thereby promote antisocial behavior.

The CDW, relative to HW, displayed reduced GMV of the left hippocampus. This group difference disappeared after adjustment for past and current anxiety and depression, past and current substance misuse, and maltreatment, which suggest that observed GMV alteration in left hippocampus was associated with past and current comorbid conditions. In line with this finding, reduced left hippocampal volumes were observed among adolescents with alcohol use disorder and comorbid CD, relative to healthy peers (Nagel, Schweinsburg, Phan, & Tapert, 2005). Even after excluding the adolescents with comorbid CD, the adolescents with alcohol use disorder alone displayed smaller left hippocampal volumes than controls suggesting that observed abnormality was associated with alcohol misuse and not with CD (Nagel et al., 2005). Reduced hippocampal volume is frequently reported in depressive disorder (Cole, Costafreda, McGuffin, & Fu, 2011; Stratmann et al., 2014), and early stress has been associated with attenuated development of the left hippocampus (Teicher et al., 2003). Adult women who experienced maltreatment in childhood display reduced GMV in the left hippocampus (Van Dam, Rando, Potenza, Tuit, & Sinha, 2014). The hippocampus is critical for the retrieval of emotional memories and contextual fear conditioning (Fanselow, 2000), and the left hippocampus is specifically activated by unpleasant emotions (Lane et al., 1997). Maltreatment-related stress may alter brain structures involved in cognition in the developing brain (Lupien, McEwen, Gunnar, & Heim, 2009), thereby further modifying the abnormal neural development associated with CD.

The CDW relative to HW showed reduced GMV in the ACC. This difference survived adjustment for maltreatment but disappeared after adjustment for past and current anxiety and depression or past and current substance misuse. A meta-analysis determined that adults with major depression and anxiety disorders presented 25% to 50% reductions in GMV in the ACC (Van Tol et al., 2010). This finding from the present study, considered in light of studies of the high prevalence of anxiety and depression among individuals with CD, again highlights the importance of taking account of these internalizing disorders when examining the neural correlates of CD. A similar reduction of GMV of the ACC was reported in adult males with CD/ASPD (Kumari et al., 2014). Several studies have identified functional impairments among male offenders during an affective memory task (Kiehl, Liddle, & Hopfinger, 2000), among men with CD/ASPD during a working memory task (Kumari et al., 2006), and among boys with CD when viewing negative emotional pictures (Sterzer, Stadler, Krebs, Kleinschmidt, & Poustka, 2005). Reduced GMV in the ACC may contribute to emotion dysregulation, and impairments in conflict monitoring and affect processing (Bush, Luu, & Posner, 2000).

The CDW, in comparison to HW, were characterized by reduced total GMV. Reductions in whole-brain GMV have been reported among boys with CD (Huebner et al., 2008), adolescent male homicide offenders (Cope, Ermer, Gaudet, et al., 2014), and adult male offenders with CD/ASPD (Barkataki, Kumari, Das, Taylor, & Sharma, 2006). In the present study, results suggested that this group difference was associated with past diagnoses of alcohol and drug dependence and anxiety and depression, and current anxiety and depression symptoms.

Based on evidence from males with CD and ASPD, we hypothesized that CDW would display alterations of GMV in the amygdala, anterior insula, and OFC relative to HW. Our hypotheses were not confirmed. The lack of group differences in amygdala, anterior insula, and OFC gray matter volumes could be due to the size of the sample. As noted above, the study was powered to detect large size effects. Thus, any abnormalities in these structures among the CDW are likely to be subtle. Other reasons for the absence of differences in these structures may be related to age and pubertal stage. Presently, there is not sufficient evidence to determine whether reductions in these structures characterize girls with CD, adult women with ASPD or psychopathy, and/or both.

Another reason that could possibly explain why we found no differences in the anterior insula and OFC may relate to low levels of callousness as indexed by PCL affective Facet 2 scores, in our sample of CD women. In a recent study of adolescent boys, volume reductions in left OFC was found to be related to high callous unemotional traits (Sebastian et al., 2016). One previous study of adolescent girls reported that those with CD, as compared to those without CD, showed reduced GMV in the bilateral anterior insula and the right striatum (Fairchild et al., 2013). However, while the group difference in the right anterior insula remained significant after controlling for callous-unemotional traits, the group difference in the left anterior insula and the striatum disappeared. The striatum difference was also associated with cannabis use. Another reason for the absence of differences in the structures could be elevated level of comorbid disorders and maltreatment in our CD sample.

Alternately, or in addition, the absence of differences in GMV of the amygdala, anterior insula, and OFC between the women with prior CD and the HW could indicate differences in the neural correlates of CD in females as compared to males as there are both structural (Gur et al., 2002) and functional (Whittle et al., 2011) brain differences between females and males. One recent meta-analysis of whole-brain structural neuroimaging studies found reduced gray matter volume in amygdala (Rogers & De Brito, 2016) among youth with conduct problems. However meta-regression analyses revealed a higher proportion of males with conduct problems in the sample was associated with decreased GMV in the left amygdala (Rogers & De Brito, 2016). Another meta-analysis that included antisocial males and females of all ages did not find differences in GMV of the amygdala and OFC (Aoki, Inokuchi, Nakao, & Yamasue, 2014). Further, abnormalities of the principal white matter tract connecting the amygdala and OFC, the uncinate fasciculus, have been observed among males and not in females with CD (Zhang et al., 2014) and similarly not in a sample of CDW that overlapped with the sample in the present study (Lindner et al., 2016). Taken together, these contradictory results highlight the importance of investigating brain correlates of CD taking account of past traumas and comorbid disorders.

There are few studies of females with antisocial behavior, and the existing studies examine very different types of samples, thereby limiting comparability of the results. One recent study reported that adolescent girls with severe substance misuse and conduct problems, relative to healthy girls, displayed reduced GMV in right dorsolateral prefrontal cortex, left ventrolateral prefrontal cortex, medial OFC, ACC, bilateral somatosensory cortex, left supramarginal gyrus, and bilateral angular gyrus (Dalwani et al., 2015). However the study selected cases with both conduct and substance misuse problems and only 14 out of 22 of these females met criteria for CD. Another study of girls detected no group differences in gray matter volumes of those with and without CD (Michalska et al., 2015). In a study of female delinquents, incarcerated in a maximum security facility, OFC, parahippocampal cortex, temporal poles, and left hippocampus gray matter volume were negatively correlated with psychopathic traits (Cope et al., 2014). However the study did not include a comparison with healthy (nonoffender) participants.

Results of the present study raise important issues. One, the results of the present study and of many others show that substance use, comorbid disorders, and maltreatment that typically characterize children with CD are each associated with distinct alterations of GMV among women with prior CD. The result of our study might indicate that frequently observed structural brain alterations in CD patients could be partly associated with past as well as current comorbid condition and maltreatment rather than being specific to CD. Consequently, in order to identify neural mechanisms associated with CD it is essential to develop methods that disentangle neural abnormalities associated with multiple comorbid disorders and maltreatment typically experienced by antisocial individuals. Using groups with and without comorbidities (Schiffer et al., 2011) and various statistical strategies (De Brito, Hodgins, Mccrory, et al., 2009), as done in the present and past studies, are not entirely satisfactory and each strategy presents disadvantages. Prospective longitudinal studies that repeatedly assess disorders and brain structures and functioning are needed.

The second issue raised by the results is that the observed alterations of GMV among the CDW may vary with age at the time of the brain scan. Although these women were age, on average 24 years, at the time of the brain scan, CD and comorbid disorders were associated with specific abnormalities. While scanning prior to puberty is unlikely to reflect the effects of chronic substance use on group differences in GMV, our results suggest that further alterations may occur as the brain matures. The results of the present study highlight the need for prospective, longitudinal investigations to identify the onset and transition of specific brain abnormalities underlying CD and comorbid disorders.

Finally, females who had CD in childhood/adolescence, most of whom did not present ASPD in adulthood, self-reported more aggressive behavior, more symptoms of anxiety and depression, and more drug use than healthy women. Fewer than half of the CDW had graduated from high school, just over half were employed or enrolled in education in the past 2 years, and almost half had given birth 10 years earlier than the average age at first birth of women in Stockholm. Additionally, in adulthood these women exhibited GMV abnormalities in several brain regions. Both the behavior patterns and the brain structure alterations shown by these women highlight the importance of intervening early to prevent childhood disorders so as to promote healthy development. Early interventions for girls presenting conduct problems may also contribute to limiting the intergenerational transfer of antisocial behavior.

### Strengths and limitations

This is the first structural neuroimaging study of women who had presented CD in childhood/adolescence that took account of past and current comorbid externalizing and internalizing disorders and maltreatment. Structured, validated, instruments were used to assess mental disorders and maltreatment, and most of the women with CD had been assessed five times since midadolescence. Prior to the scan, measures of recent alcohol/drug use and anxiety and depression symptoms were obtained. Stringent statistical thresholds were applied and differences were found in large clusters, adding confidence to findings. However our findings should be viewed in the context of elevated levels of psychiatric comorbidity and history of maltreatment amongst CDW. Such diagnostic comorbidity presents a challenge to identify brain abnormalities associated with specifically with CD. Nevertheless comorbidity in disruptive behavior disorders is a rule rather than an exception. Recruiting subjects with only diagnosis of CD may allow easy interpretability of results but limits the generalizability of the findings. Although women with CD in our sample presented comorbid psychopathology that reduces the ease of interpretation of results, yet this make them more representative of clinical samples of females with CD than a “pure” group would have been. Importantly, given that females with CD present high levels of comorbid substance misuse, anxiety and depression disorders, and maltreatment, group comparisons of GMV were rerun to investigate associations with each covariate. A weakness of the study is the small sample size that resulted primarily from the difficulty of following antisocial females. The sample size limited detection of group differences that were small and intermediate in effect size.

Further, the small sample size and cross-section design did not allow us to examine how CD and other aspects of psychopathology interact with each other over time to affect overall brain structure. While our findings are exploratory and require replication, they do point out the importance of taking account of comorbid disorders and maltreatment in research aimed to further the understanding of CD.

While we had no measure of head injuries that may have resulted from engagement in risky behaviors, no participant reported loss of consciousness for more than 30 minutes.

## Conclusions

At an average age of 24 years, women who had presented CD in childhood/adolescence, relative to the healthy women displayed abnormalities of GMV in left STG, lingual gyrus, left hippocampus, and left ACC that were associated with past comorbid disorders, maltreatment, and current substance use and anxiety and depression symptoms. Further, the women with CD were characterized by reduced whole-brain GMV that was also associated with past comorbid disorders. Results highlight the importance of taking account of comorbid internalizing disorders, substance use, and a history of maltreatment in studies of the neural correlates of CD.
